# Fault Diagnosis of a Bogie Gearbox Based on Pied Kingfisher Optimizer-Improved Complete Ensemble Empirical Mode Decomposition with Adaptive Noise, Improved Multi-Scale Weighted Permutation Entropy, and Starfish Optimization Algorithm–Least-Squares Support Vector Machine

**DOI:** 10.3390/e27090905

**Published:** 2025-08-26

**Authors:** Guangjian Zhang, Shilun Ma, Xulong Wang

**Affiliations:** School of Automobile and Transportation, Tianjin University of Technology and Education, Tianjin 300222, China; 2020040002@tute.edu.cn (S.M.); xulong.wang@tute.edu.cn (X.W.)

**Keywords:** bogie gearbox, fault diagnosis, PKO-ICEEMDAN, IMWPE, SFOA-LSSVM

## Abstract

Current methods of detecting bogie gearbox faults mainly depend on manual judgment, which leads to inaccurate fault identification. In this study, a fault diagnosis model is proposed based on a pied kingfisher optimizer-improved complete ensemble empirical mode decomposition with adaptive noise (PKO-ICEEMDAN), improved multi-scale weighted permutation entropy (IMWPE), and a starfish optimization algorithm optimizing a least-squares support vector machine (SFOA-LSSVM). Firstly, the acceleration signals of a bogie gearbox under six different working conditions were extracted through experiments. Secondly, the acceleration signals were decomposed by ICEEMDAN optimized by PKO to obtain the intrinsic mode function (IMF). Thirdly, IMFs with rich fault information were selected to reconstruct the signals according to the double screening criteria of both the correlation coefficient and variance contribution rate, and the IMWPE of the reconstructed signals was extracted. Finally, IMWPE as a feature vector was input into LSSVM optimized by the SFOA for fault diagnosis and compared with various models. The results show that the average accuracy of the training data of the proposed model was 99.13%, and the standard deviation was 0.09, while the average accuracy of the testing data was 99.44%, and the standard deviation was 0.12. Thus, the effectiveness of the proposed fault diagnosis model for the bogie gearbox was verified.

## 1. Introduction

A bogie gearbox is an important mechanical transmission mechanism of a rail train, which is of great significance to the safe operation of the train [1]. As the mechanical rotating part of the bogie, the health monitoring of the gearbox is extremely important [2]. In terms of the current preventive maintenance scheme, it is very difficult to find relevant faults in a bogie gearbox in time [3]. Therefore, once the gearbox breaks down during train operation, it will cause heavy losses that are difficult to estimate [4]. Today, the rise of intelligent algorithms has introduced a new perspective to the fault diagnosis of the mechanical structure of rail trains, and numerous scholars have also carried out fruitful work [5,6].

For the signal pre-processing method, the authors of [7] adopted empirical mode decomposition (EMD) to decompose the vibration signals of the gearbox to obtain the intrinsic mode function (IMF). Although the classification effect was good, the EMD algorithm had some defects, such as mode mixing and end effects. Based on this, the authors of [8] used an extended EMD to decompose the vibration signals of the gearbox. Although the end effect of the signal decomposition was eliminated, the phenomenon of mode mixing could not be avoided. Therefore, the authors of [9] used ensemble empirical mode decomposition (EEMD) to decompose the vibration signals of the gearbox. Although the EEMD added white noise to the original signals and offset the white noise by the ensemble average, it still remained white noise and affected the calculation results. Subsequently, the authors of [10,11] employed complementary ensemble empirical mode decomposition (CEEMD) to handle signals to obtain the IMF. However, if the amplitude and iteration times of the added white noise were not properly selected, redundant IMF components would be generated, which would affect the subsequent calculation results. The authors of [12,13] utilized complete ensemble empirical mode decomposition with adaptive noise (CEEMDAN) to analyze the vibration signals of rolling bearings. Although CEEMDAN added adaptive noise, it still had the problems of mode interference and data instability. On this foundation, the authors of [14] made use of an improved complete ensemble empirical mode decomposition with adaptive noise (ICEEMDAN) to process the vibration signals of gearbox bearings. Unlike the CEEMDAN algorithm, which directly added white noise during signal decomposition, a good fault diagnosis effect was obtained by adding Gaussian white noise decomposed by EMD. However, the two parameters of the white noise amplitude weight *Nstd* and noise addition times *NE* in the ICEEMDAN algorithm will affect its performance, so they need to be optimized by an optimization algorithm.

Entropy is often regarded as the eigenvalue of a system for fault diagnosis [15]. Fuzzy entropy (FE) [16], dispersion entropy (DE) [17], sample entropy (SE) [18], power spectral entropy (PSE) [19], permutation entropy (PE) [20], and others have been widely used. The permutation entropy of a gearbox was extracted in [21] for fault diagnosis. The permutation entropy had a strong anti-noise capability and fast calculation speed, which showed its advantages to a certain extent. However, the permutation entropy ignored the amplitude information of time series. Therefore, the authors of [22] used the variance as the weight and introduced weighted permutation entropy (WPE) to verify the effectiveness of the model. However, the WPE still only processed data at a single timescale. Based on this, the authors of [23] used multi-scale weighted permutation entropy (MWPE) as the eigenvalue of the motor for fault diagnosis, which surmounted the shortcomings of the WPE with single-timescale data analysis. However, the error of the MWPE in coarse graining processes will increase with the increase in the scale factor. Hence, the authors of [24] further improved on the basis of the MWPE, averaging the entropy of multiple coarse-grained sequences at the same scale, and obtaining more accurate results.

There are many kinds of fault diagnosis classifiers. The authors of [25] used a probabilistic neural network (PNN) to diagnose the faults of rolling bearings. However, the PNN needed more memory to store kernel functions, and it needed higher computing resources for large-scale data. The authors of [26] applied long short-term memory (LSTM) to diagnose the faults of a distributed elevator; however, the LSTM model needed a large number of samples for model training, and there was high computational complexity. The authors of [27] utilized recurrent neural networks (RNNs) to diagnose the faults of three-phase induction motor. However, there were the defects of long dependence, gradient disappearance, and gradient explosion in the RNN. The authors of [28] used a support vector machine (SVM) to diagnose the faults of rolling bearings. Because there were inequality constraints in the SVM, it had the limitation of a large amount of calculation. The authors of [29] adopted a least-squares support vector machine (LSSVM) to diagnose the faults of rolling bearings. In the LSSVM, equality constraints were used to replace inequality constraints, improving the classification effect, but the penalty factor *δ* and kernel function parameter *θ* needed to be set manually.

Based on the above research, the goal of this study was to provide an effective intelligent algorithm for fault diagnosis of a bogie gearbox. The vibration acceleration signals of a bogie gearbox under six working conditions were collected via a certain type of rail vehicle test-bed in this study. IMF was obtained through ICEEMDAN decomposition signals. In order to find the best combination of ICEEMDAN parameters, pied kingfisher optimizer (PKO) [30] was used for optimization. IMF was optimized based on the double screening criteria—correlation coefficient and variance contribution rate—and the signals were reconstructed. Improved multi-scale weighted permutation entropy (IMWPE) of the reconstructed signals was obtained. IMWPE was input as a feature vector into LSSVM for fault diagnosis. At the same time, the starfish optimization algorithm (SFOA) [31] was used to optimize the penalty factor and kernel function parameter of LSSVM to obtain the best combination of parameters. The validity of the model was verified by comparing the performance evaluation indices of various models.

## 2. Relevant Theories

### 2.1. Pied Kingfisher Optimizer (PKO)

Inspired by the predatory behavior of the pied kingfisher in nature, PKO is a new meta-heuristic algorithm, which is divided into four stages: initialization, exploration, development, and symbiosis.

(1) Initialization Stage:

In the search space, PKO randomly generates a set of initial solutions to start the search process. The mathematical expression is

(1) $$Y_{i,j}=S_{l}+(S_{u}-S_{l})\times r,i=1,2,\ldots ,N\&j=1,2,\ldots ,M$$

where *Y_i_*_,*j*_ is the position of the *i*th individual in the *j*th dimension, *r* is a random number between 0 and 1, *S_l_* is the lower limit of the search space, *S_u_* is the upper limit of the search space, *N* is the population size, and *M* is the problem dimension.

(2) Exploration Stage:

In the exploration stage of PKO, the mathematical expression of the position of the pied kingfisher is

(2) $$\left\{\begin{array}{l} Y_{i}(t+1)=Y_{i}(t)+\beta \times A\times [Y_{j}(t)-Y_{i}(t)]\\ \beta =2\times R(1,M) \end{array}\right.$$

where *Y_i_* (*t* + 1) and *Y_i_* (*t*) represent the position of the next iteration and the current iteration of pied kingfisher, respectively; *t* represents the current number of iterations; *i* and *j* are integers from 1 to *N,* and *i* is not equal to *j*; and *R* is a random number in normal distribution. The parameter *A* is dynamically adjusted according to the “perching” or “hovering” strategies of pied kingfishers to ensure the best performance.

(1) In the perching strategy, the mathematical model of *A* is

(3) $$\left\{\begin{array}{l} A=\left\{\exp(1)-\exp\left[{\left(\frac{t-1}{T_{\max}}\right)}^{1/E}\right]\right\}\times \cos B\\ B=2\times \pi \times r \end{array}\right.$$

where *T*_max_ represents the maximum number of iterations, *E* is the jumping factor (with a value of 8), and *B* is the crest feather angle of the pied kingfisher.

(2) In the hovering strategy, the mathematical model of *A* is

(4) $$A=r\times \frac{G(j)}{G(i)}\times \frac{t^{1/E}}{{\left(T_{\max}\right)}^{1/E}}$$

where *G*(*j*) and *G*(*i*) are the fitness values of the *j*th and *i*th pied kingfisher, respectively.

(3) Development Stage:

The mathematical model of the pied kingfisher’s diving predation strategy is

(5) $$Y_{i}(t+1)=Y_{i}(t)+K\times d\times h\times [p-Y_{\mathrm{best}}(t)]$$

(6) $$\left\{\begin{array}{l} K=r\times [G(i)/G_{\mathrm{best}}(i)]\\ \varepsilon =\exp{\left[(-t)/T_{\max}\right]}^{2}\\ \eta =2\times R(1,M)-1\\ q=Y_{i}(t)+\delta^{2}\times R\times Y_{\mathrm{best}}(t) \end{array}\right.$$

where *G*_best_(*i*) is the best fitness value, *K* and *ε* represent hunting ability, *η* is the control parameter, and *q* is the flapping frequency of the pied kingfisher’s wings.

(4) Symbiosis Stage:

The pied kingfisher is symbiotic with a variety of animals; the mathematical model is

(7) $$Y_{i}(t+1)=\left\{\begin{array}{l} Y_{u}(t)+\varepsilon \times \eta \times \left|Y_{i}(t)-Y_{v}(t)\right|,r>(1-XL)\\ Y_{i}(t),\mathrm{else} \end{array}\right.$$

(8) $$XL=XL_{\max}-(XL_{\max}-XL_{\min})\times (t/T_{\max})$$

where *Y_u_* and *Y_v_* are two individual positions randomly selected from the population of pied kingfishers, and *XL* is the predation efficiency; *XL*_max_ = 0.5; *XL*_min_ = 0.

### 2.2. Improved Complete Ensemble Empirical Mode Decomposition with Adaptive Noise (ICEEMDAN)

Based on the EMD decomposition method, ICEEMDAN further improves the CEEMDAN decomposition principle. Unlike the CEEMDAN decomposition principle, ICEEMDAN defines the IMF as the difference between residual signals and the local mean, and its algorithm steps are as follows:

(1) Construct *M* noise-controllable signals.

(9) $$\left\{\begin{array}{l} X_{1}^{\left(m\right)}=x+\mu_{1}F_{1}(g^{\left(m\right)})\\ \mu_{1}=\gamma_{1}\sigma (x)/\sigma (F_{1}(g^{\left(m\right)})) \end{array}\right.(m=1,2,\ldots ,M)$$

where *x* represents the original signals, *g*^(*m*)^ is the Gaussian white noise, *μ*_1_ is the expected signal-to-noise ratio, *γ*_1_ is the amplitude, *σ* (·) is the mathematical expectation operator, and *F*_1_ (·) represents the operator of the first IMF component obtained by EMD decomposing the signals.

(2) Calculate the difference between each $X_{1}^{\left(m\right)}$ and its first IMF, and compute the average of *M* differences. The residual *a*_1_ of the first decomposition is as follows:

(10) $$a_{1}=\langle X_{1}^{\left(m\right)}-F_{1}(X_{1}^{\left(m\right)})\rangle$$

where <·> is the operator for calculating the average of *M* signals.

(3) The original signals *x* minus the first residual *a*_1_. Obtain the first IMF component *b*_1_ of the original signals.

(11) $$b_{1}=x-a_{1}$$

(4) When *n* ≥ 2, construct the *n*th group of *M* noise-controllable signals.

(12) $$X_{n}^{\left(m\right)}=a_{n-1}+\mu_{n}F_{n}(g^{\left(m\right)}),(m=1,2,\ldots ,M)$$

(5) Calculate the residual *a_n_* of the *n*th decomposition.

(13) $$a_{n}=\langle X_{n}^{\left(m\right)}-F_{n}(X_{n}^{\left(m\right)})\rangle$$

(6) The last residual *a_n_*_−1_ minus the residual *a_n_* to obtain the *n*th IMF component *b_n_* of the original signals.

(14) $$b_{n}=a_{n-1}-a_{n}$$

(7) Let *n* = *n* + 1, and return to Step 4 to calculate the next *n* value. The calculation can be terminated until the residual meets the iteration conditions: ① Meet residual *b_n_* monotonicity. ② The Cauchy convergence criterion is satisfied; that is, the standard deviation *std* between two adjacent IMF components is less than a limit value.

(15) $$std={\left\|b_{n}\;-\;b_{n-1}\right\|}_{2}/{\left\|b_{n}\right\|}_{2}$$

### 2.3. Improved Multi-Scale Weighted Permutation Entropy (IMWPE)

(1) Weighted Permutation Entropy (WPE)

The WPE algorithm’s steps are as follows:

(1) For the original time series ***Y*** = {*y*(*i*), *i* = 1, 2,…, *N*}, $Y_{i}^{\left(m\right)}$ is reconstructed in phase space.

(16) $$Y_{i}^{\left(m\right)}=\{y_{i}^{\left(m\right)},y_{i+\tau }^{\left(m\right)},\ldots ,y_{i+(m-1)\tau }^{\left(m\right)}\}$$

where *m* is the embedding dimension, and *τ* is the time delay.

(2) Calculate the weight *w_i_* of the sub-signals:

(17) $$\left\{\begin{array}{l} w_{i}=(1/m)\sum_{k=1}^{m}{\left[y_{i+(k-1)\tau }-{\bar{Y}}_{k}^{\left(m\right)}\right]}^{2}\\ {\bar{Y}}_{k}^{\left(m\right)}=(1/m)\sum_{k=1}^{m}y_{i+(k-1)\tau },k=1,2,\ldots ,m \end{array}\right.$$

(3) Calculate the probability of each arrangement. The characteristic information of the sub-signals $Y_{i}^{\left(m\right)}$ is characterized by *w_i_* and the sorting mode π*_k_*. There are *k* sorting modes in signals ***Y***. The probability *P_w_*(π*_k_*) of occurrence of each sorting mode is as follows:

(18) $$P_{w}(\pi_{k})=\frac{\sum \left[w_{i}\left|1\leq i\leq N-(m-1)\right.\tau ,i\in Z^{+},N(Y_{i}^{\left(m\right)})\right]}{\sum w_{i}}$$

where *N*($Y_{i}^{\left(m\right)}$) is the sorting mode π*_k_* of $Y_{i}^{\left(m\right)}$.

(4) Calculate the weighted permutation entropy *E*_WPE_ of the signals:

(19) $$E_{\mathrm{WPE}}(Y,m,\tau )=-\frac{1}{\ln(m!)}\sum_{k=1}^{K}P_{w}(\pi_{k})\ln P_{w}(\pi_{k})$$

(2) Multi-Scale Weighted Permutation Entropy (MWPE)

The steps of the MWPE algorithm are as follows:

(1) The original signals ***Y*** = {*y*(*i*), *i* = 1, 2,…, *N*} are subjected to a coarse graining process to obtain the coarse-grained sequence $z_{j}^{\left(l\right)}$.

(20) $$z_{j}^{\left(l\right)}=\frac{1}{l}\sum_{i=(j-1)l+1}^{jl}y(i),1\leq j\leq \frac{N}{l}$$

where *l* is the scale factor.

(2) The weighted permutation entropy *E*_MWPE_ of each coarse-grained sequence is as follows:

(21) $$E_{\mathrm{MWPE}}(Y,m,\tau ,l)=E_{\mathrm{WPE}}[z^{\left(l\right)},m,\tau ]$$

(3) Improved Multi-Scale Weighted Permutation Entropy (IMWPE)

The IMWPE algorithm’s steps are as follows:

(1) The signal is processed by improved coarse-grained processing to generate *l* new sequences:

(22) $$\left\{\begin{array}{l} U_{k}^{\left(l\right)}=\{u_{k,j_{1}}^{\left(l\right)},u_{k,j_{2}}^{\left(l\right)},\ldots ,u_{k,j_{l}}^{\left(l\right)}\}\\ u_{k,j}^{\left(l\right)}=\frac{1}{l}\sum_{i=(j-1)l+k}^{jl+k-1}y(i),1\leq j\leq \frac{N}{l},1\leq k\leq l \end{array}\right.$$

(2) WPE is calculated and averaged for the coarse-grained sequence $U_{k}^{\left(l\right)}$ for the scale factor *l* to obtain the IMWPE:

(23) $$E_{\mathrm{IMWPE}}(Y,m,\tau ,l)=(1/l)\sum_{k=1}^{l}E_{\mathrm{WPE}}[U_{k}^{\left(l\right)},m,\tau ]$$

**Figure 1:**
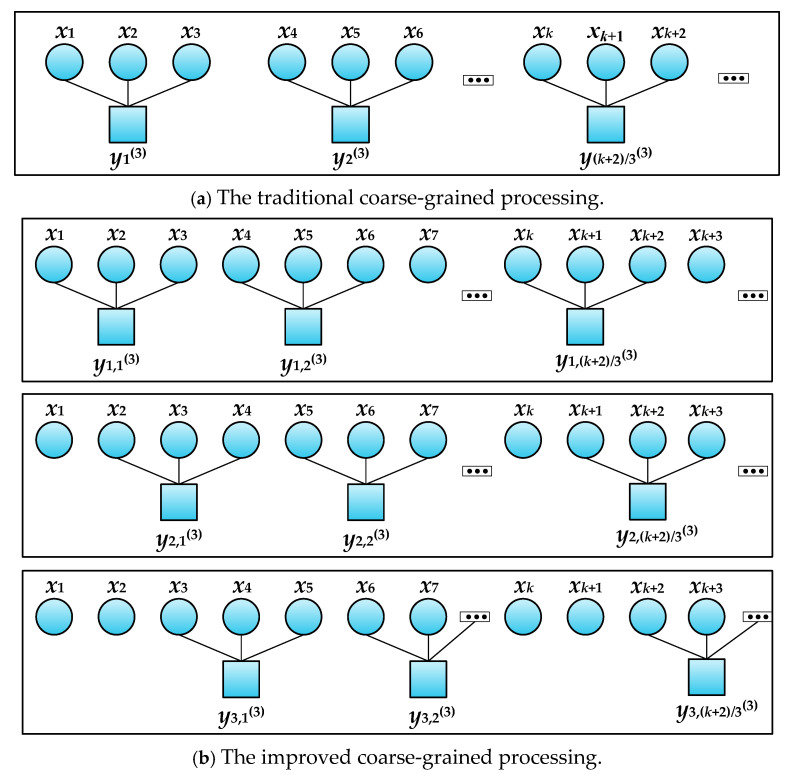
Schematic diagram of traditional and improved coarse-grained processing (*l* = 3).

When the scale factor is equal to 3, the traditional coarse-grained processing and the improved coarse-grained processing are as shown in Figure 1.

### 2.4. Starfish Optimization Algorithm (SFOA)

Inspired by the predation behavior of starfish in the ocean, the SFOA is divided into an initialization stage, exploration stage, and development stage.

(1) Initialization Stage:

In the initialization stage, the starfish population randomly generates the position, and its mathematical expression is as follows:

(24) $$X_{ij}=L_{j}+R\times (U_{j}-L_{j}),i=1,2,\ldots ,N\&j=1,2,\ldots ,M$$

where *X_ij_* is the *j*th dimensional position of the *i*th starfish, *R* is a random number between 0 and 1, *U_j_* is the upper limit of the *j*th dimensional design variable, *L_j_* is the lower limit of the *j*th dimensional design variable, *N* is the population number, and *M* is the problem dimension.

(2) Exploration Stage:

A starfish has five arms. A new search mode is proposed in the exploration stage. The 5-dimensional search mode is combined with the 1-dimensional search mode.

(1) If the dimension of the optimization problem is greater than 5, the starfish moves five arms to search for food. The mathematical expression is as follows:

(25) $$\left\{\begin{array}{l} Y_{i,p}(t)=X_{i,p}(t)+\alpha_{1}[X_{\mathrm{best},p}(t)-X_{i,p}(t)]\cos\varphi ,R\leq 0.5\\ Y_{i,p}(t)=X_{i,p}(t)-\alpha_{1}[X_{\mathrm{best},p}(t)-X_{i,p}(t)]\sin\varphi ,R>0.5\\ \alpha_{1}=2(R-1)\times \pi \\ \varphi =(\pi /2)\times (t/T_{\max}) \end{array}\right.$$

where *Y_i_*_,*p*_(*t*) represents the position obtained by the starfish, *X_i_*_,*p*_(*t*) represents the current position of the starfish, *X*_best,*p*_(*t*) represents the *p*th dimension of the current best position, *p* is five randomly selected dimensions in the *M* dimension, *φ*∈[0, π/2], and *T*_max_ is the maximum number of iterations.

(2) If the dimension of the optimization problem is less than 5, the starfish uses a 1-dimensional model to search, and its mathematical model is as follows:

(26) $$\left\{\begin{array}{l} Y_{i,p}(t)=EN\times X_{i,p}(t)+B\times [X_{y,p}(t)-X_{i,p}(t)]+C\times [X_{z,p}(t)-X_{i,p}(t)]\\ EN=(T_{\max}-t)/(T_{\max})\cos\varphi \end{array}\right.$$

where *X_y_*_,*p*_(*t*) and *X_z_*_,*p*_(*t*) are the *p*-dimensional positions of two starfish randomly selected in the population, *B*, *C*∈[−1, 1], and *EN* is the energy of the starfish.

(3) Development Stage:

In the development stage, the starfish implements two strategies: predation and regeneration.

(1) Predation Strategy: The starfish uses a parallel bidirectional search method with the information of other starfishes and the best position of the current population. First, the five distances between the best position and other starfishes are calculated, and then two distances are randomly selected for confirmation, so as to update the starfish population. The distance expression is as follows:

(27) $$D_{m}=[X_{\mathrm{best}}(t)-X_{m_{p}}(t)],m=1,2,3,4,5$$

where *D_m_* is the distance between the five global best starfishes obtained and other starfishes, *m_p_* represents five randomly selected starfishes, and the update rule of each starfish is as follows:

(28) $$Y_{i}(t)=X_{i}(t)+R_{1}D_{m1}+R_{2}D_{m2}$$

where *R*_1_ and *R*_2_ are random numbers between 0 and 1, and *D_m_*_1_ and *D_m_*_2_ are randomly selected values in *D_m_*.

(2) Regeneration Strategy: If a starfish is captured by natural enemies, it will cut off one arm in order to escape. Therefore, the regeneration strategy is implemented in the last starfish in the population. Its mathematical expression is as follows:

(29) $$Y_{i}(t)=\exp[(-t\times N)/T_{\max}]X_{i}(t)$$

If the position of the starfish exceeds the boundary, the position expression is as follows:

(30) $$X_{i}(t+1)=\left\{\begin{array}{l} Y_{i}(t),l_{b}\leq Y_{i}(t)\leq u_{b}\\ l_{b},Y_{i}(t)<l_{b}\\ u_{b},Y_{i}(t)>u_{b} \end{array}\right.$$

### 2.5. Least-Squares Support Vector Machine (LSSVM)

Support vector machine is improved, and the inequality constraint is replaced by the equality constraint to obtain the least-squares support vector machine, avoiding the defects of the common support vector machine, which takes up a lot of computing space and produces an unsatisfactory classification effect. The mathematical expression of LSSVM is as follows:

(31) $$\left\{\begin{array}{l} \min J(\omega ,\xi )=0.5{\left\|\omega \right\|}^{2}+0.5\delta \sum_{i=1}^{n}\xi_{i}^{2}\\ s.t.y_{i}[\omega^{T}\omega (x_{i})+b]-1+\xi_{i}=0 \end{array}\right.$$

where *δ* is the penalty factor, *ξ_i_* is the error amount, *ω* represents the weight vector, *b* represents the bias term, *x_i_* is the input vector, *y_i_* is the output tag, and *J*(*ω*,*ξ*) represents the objective function.

The Lagrange function is established, and its expression is as follows:

(32) $$L(\omega ,b,\xi ,\alpha )=0.5{\left\|\omega \right\|}^{2}+0.5\delta \sum_{i=1}^{n}\xi_{i}^{2}+\sum_{i=1}^{n}\alpha_{i}(y_{i}-\omega^{T}\varphi (x_{i})-b-\xi_{i}^{2})$$

Solve the following expression:

(33) $$\left\{\begin{array}{l} \frac{\partial L}{\partial \omega }=0\Rightarrow \omega =\sum_{i=1}^{n}\alpha_{i}\varphi (x_{i})\\ \frac{\partial L}{\partial b}=0\Rightarrow \sum_{i=1}^{n}\alpha_{i}=0\\ \frac{\partial L}{\partial \xi_{i}}=0\Rightarrow \alpha_{i}=\delta \xi_{i}\\ \frac{\partial L}{\partial \alpha }=0\Rightarrow \omega \cdot \varphi (x)+b+\xi_{i}+y_{i}=0 \end{array}\right.$$

The classification function of LSSVM is obtained through arranging

(34) $$f(x)=\sum_{i=1}^{n}\alpha_{i}^{*}y_{i}K(x_{i},x_{j})+b^{*}$$

where *K*(*x_i_*, *x_j_*) is the kernel function.

In LSSVM, there are mainly four kinds of kernel functions:

(1) Linear kernel function: $K(x_{i},x_{j})=x_{i}\cdot x_{j}$;
(2) Polynomial kernel function: $K(x_{i},x_{j})={\left(x_{i}\cdot x_{j}+b\right)}^{d}$;
(3) Radial basis kernel function (RBF): $K(x_{i},x_{j})=\exp[-{\left\|x_{i}-x_{j}\right\|}^{2}/(2\theta^{2})]$;
(4) Hyperbolic tangent kernel function: $K(x_{i},x_{j})=\tanh[v(x_{i},x_{j})+c]$.

At present, there is no clear regulation on the choice of kernel function. According to a large number of experiments, the linear kernel function has good performance in linear separable problems. However, the polynomial kernel function has relatively many parameters, which can easily cause overfitting, and it is often applied to scenarios with low feature data dimensions. The hyperbolic tangent kernel function is widely used in processing symmetric data. The RBF is the most widely used kernel function, especially in nonlinear high-dimensional mapping problems. Therefore, the RBF is selected in this paper.

(35) $$K(x_{i},x_{j})=\exp[-{\left\|x_{i}-x_{j}\right\|}^{2}/(2\theta^{2})]$$

where *θ* represents the kernel function parameter.

## 3. Experiments and Data Analysis

### 3.1. PKO and SFOA Simulation Experiments

**Figure 2:**
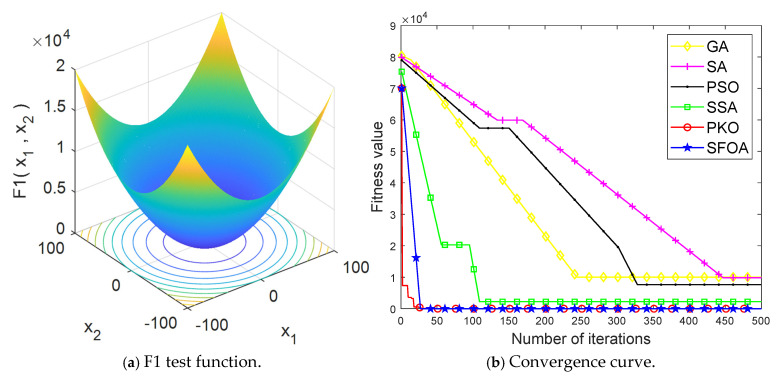
Simulation results.

In order to verify the good convergence characteristics of PKO and SFOA, the F1 function in the CEC2005 function set was selected for testing. The F1 function is a unimodal function, which is very challenging for verifying the convergence ability of the algorithm. Compared with those of the genetic algorithm (GA), simulated annealing algorithm (SA), particle swarm optimization (PSO), and sparrow search algorithm (SSA), the operation results are shown in Figure 2.

The image was magnified for observation. As shown in Figure 2, SFOA and PKO converge faster than other algorithms—SFOA converges around the 27th generation, while PKO converges around the 28th generation—and the optimization value of both is very close to the minimum value of the F1 function after many tests. The good convergence ability of the two optimization algorithms is therefore verified.

### 3.2. Gearbox Data Acquisition Experiment

**Figure 3:**
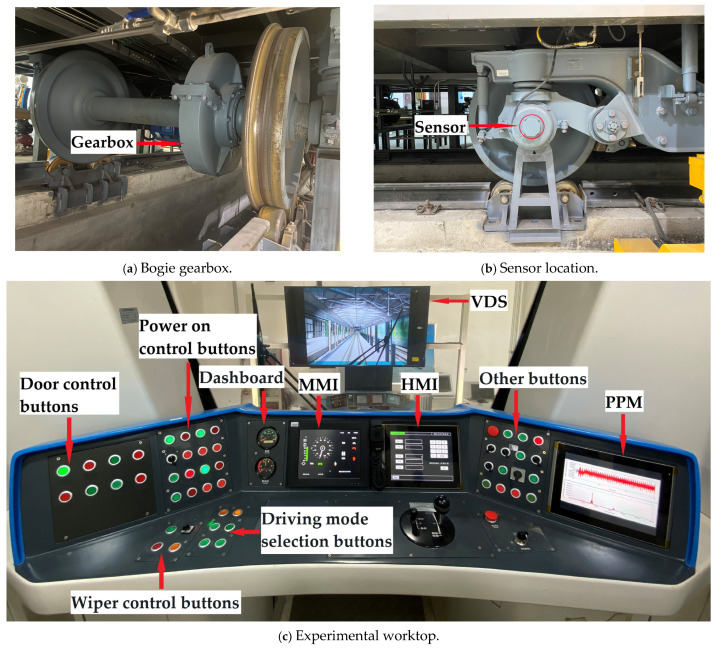
Experimental operating environment.

The vibration signal acquisition experiment on the bogie gearbox was carried out in the rail vehicle laboratory. Taking a certain type of metro vehicle as the research object, the experimental environment is shown in Figure 3. The bogie was successively installed with a gearbox under 6 working conditions, and the specific description of the gearbox under various working conditions is shown in Table 1. The sampling frequency was set to 5000 Hz, and the rated load was applied at 300 kN. The acceleration signals of the gearbox at a train speed of 80 km/h were extracted under different working conditions. In total, 200 groups of samples were collected for each working condition, with a total of 1200 groups of samples.

**Figure 4:**
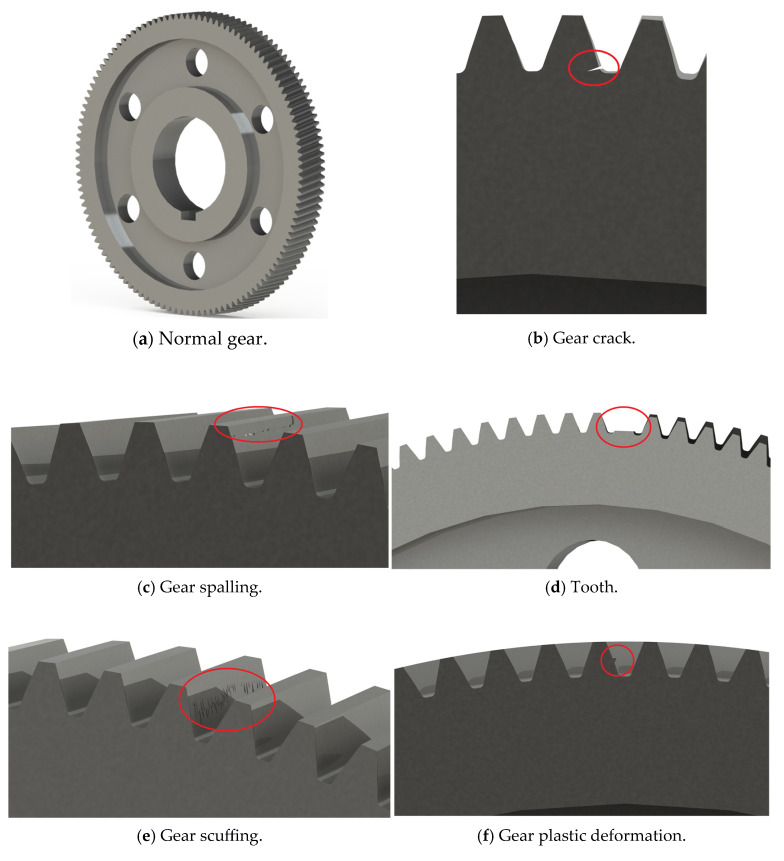
The gear status under 6 working conditions.

It can be seen from Figure 3 that the gearbox was installed on the axle of the bogie, including a driving small helical gear and a driven large helical gear. The vibration sensor was installed on the axle bearing. The relevant gear parameters are shown in Table 2. The gear status under 6 working conditions is shown in Figure 4. A human–machine interface (HMI) was used to control the train and to display various information and parameters during its operation. A post-process module (PPM) was used to process the acceleration signals of the gearbox, while a visual display system (VDS) was used to display the scene during train operation.

**Figure 5:**
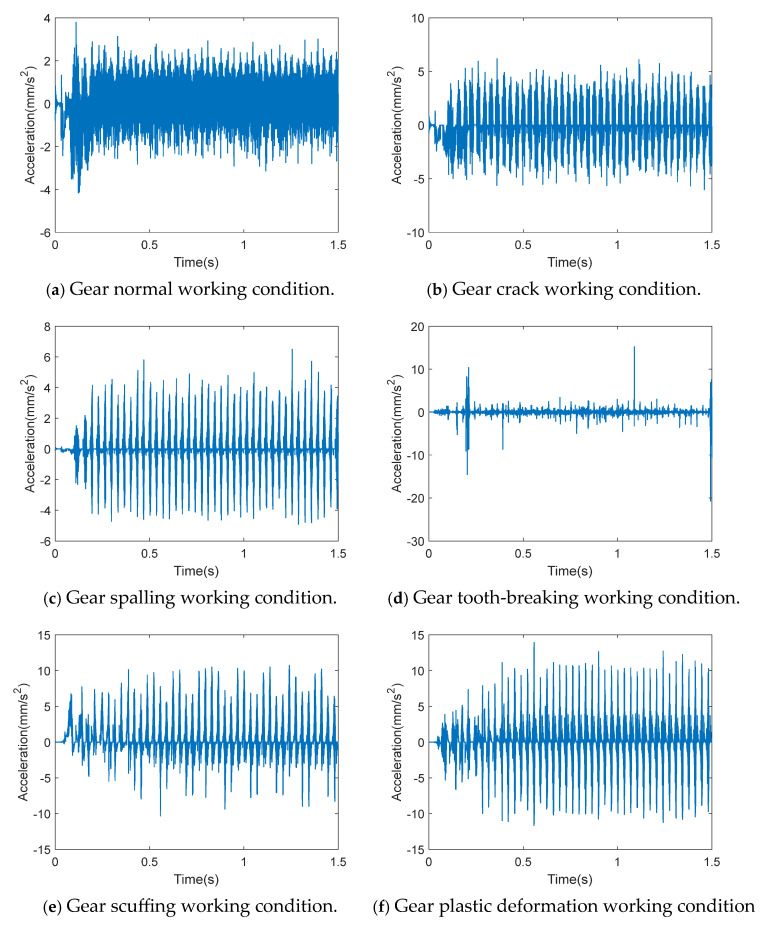
Time-domain waveform of gearbox acceleration under various working conditions.

The collected sample data were processed to obtain the time-domain waveform of gearbox acceleration under various working conditions, as shown in Figure 5.

It can be seen from Figure 5 that the vibration of the gearbox was stable under normal operating conditions. However, the waveform shows obvious protrusion under fault conditions. The vibration and impact were especially more severe under the condition of tooth-breaking, because the fit of one gear tooth was directly reduced.

### 3.3. Signals Decomposition Based on PKO-ICEEMDAN

**Figure 6:**
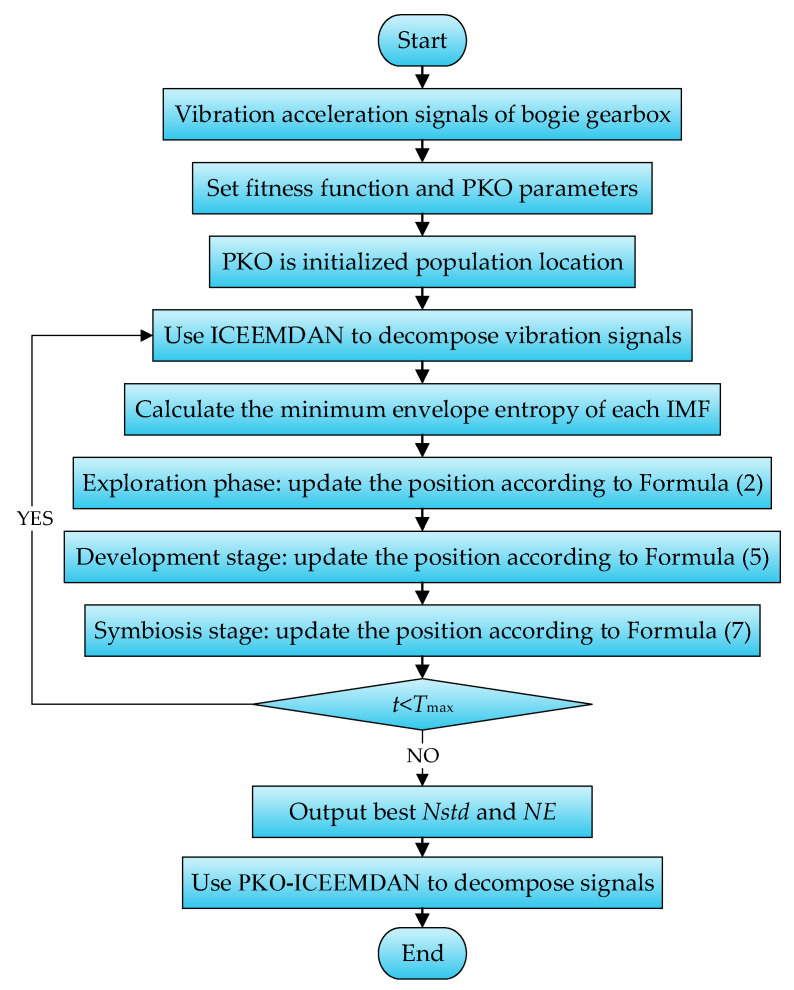
Flowchart of PKO-ICEEMDAN.

White noise amplitude weight (*Nstd*) and noise addition times (*NE*) in ICEEMDAN are 2 important parameters that affect the performance of the algorithm; they are set manually when used alone. However, artificially set parameters cannot maximize the performance of the algorithm. Therefore, the PKO algorithm was adopted to optimize these 2 parameters, and the minimum envelope entropy was used as the fitness function. The process of PKO optimizing ICEEMDAN is shown in Figure 6.

It can be seen from Figure 6 that the steps for PKO optimizing ICEEMDAN are as follows:

(1) The population size of PKO is set to 30, and the maximum number of iterations is set to 50; *Nstd* ∈ [0.2, 0.8]. *NE* ∈ [30, 1800].
(2) Calculate the fitness function to obtain the minimum envelope entropy *E_p_* and the best parameter combination. The *E_p_* calculation formula is as follows:

(36) $$E_{p}=-\sum_{i=1}^{k}p_{i}lgp_{i},p_{i}=b(i)/\sum_{i=1}^{k}b(i)$$

(3) Update the position of each stage according to the change in fitness.
(4) The iteration is terminated if the iteration condition is met, and the optimal parameter combination is output. Otherwise, the fitness function is recalculated for the next iteration.
(5) Gearbox vibration signals are decomposed by ICEEMDAN configured with the best parameter combination.
(6) The decomposed IMF satisfies 2 conditions: (1) The number of extreme points and zero crossings in a function must be equal or at most differ by one. (2) The average value of the upper envelope formed by the local maximum point and the lower envelope formed by the local minimum point of the function is zero. With these constraints and iterative conditions, the number of ICEEMDAN decomposition layers is automatically completed by the program.

**Figure 7:**
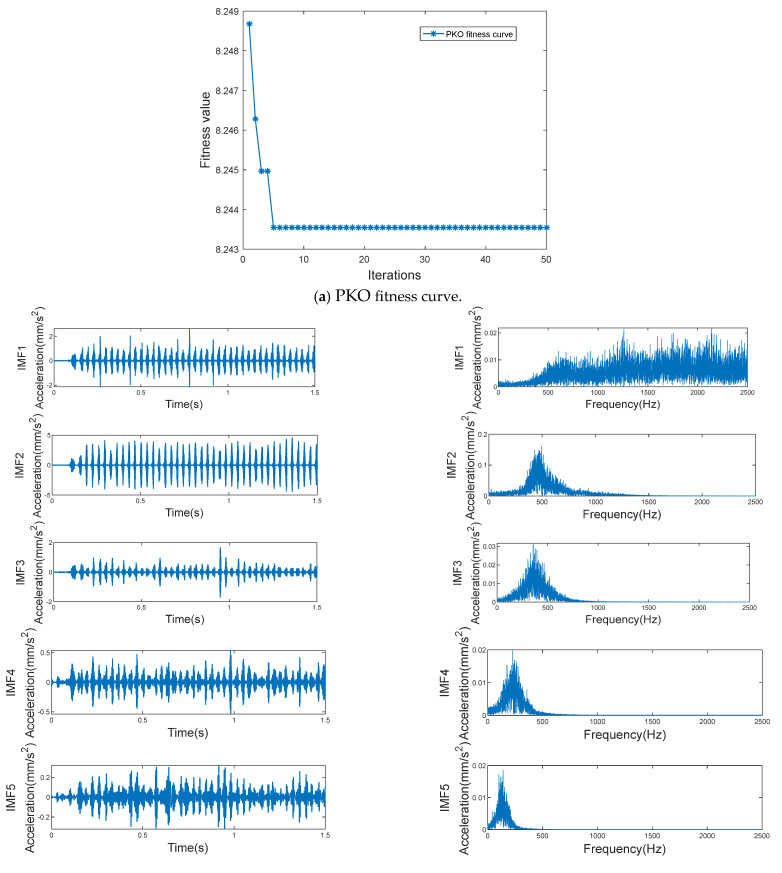

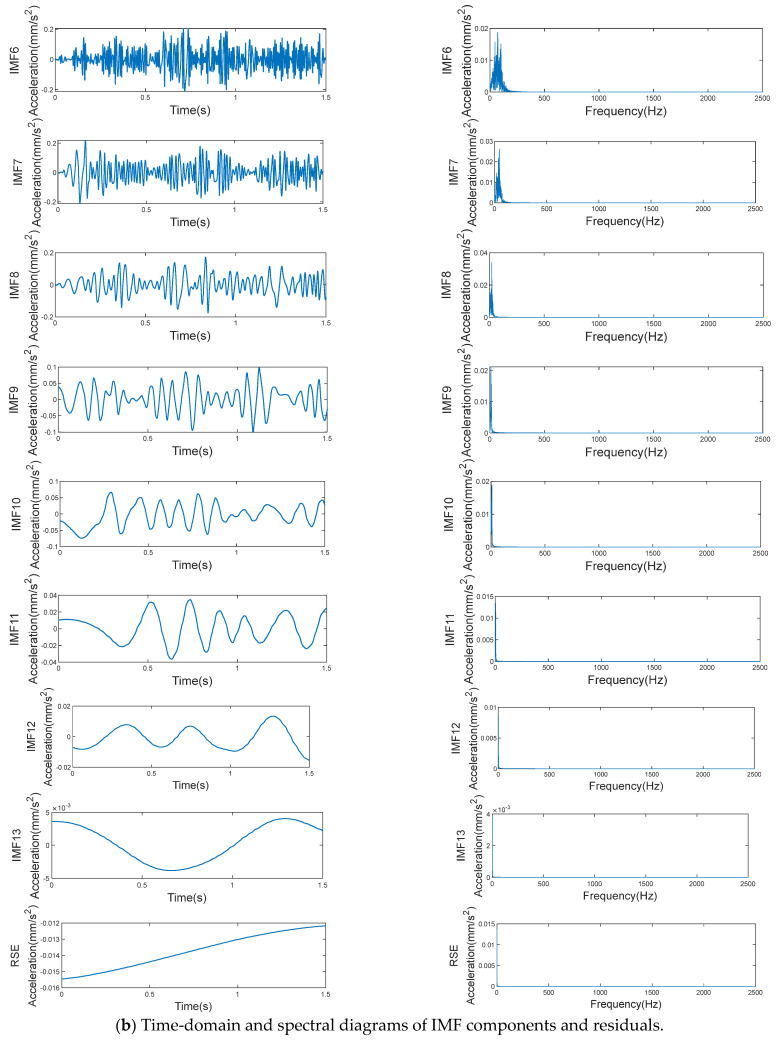
Signal decomposition effects of PKO-ICEEMDAN.

Each category of sample data was run 5 times to calculate the average of the white noise amplitude weight and noise addition times, which was the optimal parameter combination [*Nstd*, *NE*]. Then, this combination was applied to ICEEMDAN. The optimal parameter combination of gearbox data under each working condition is shown in Table 3. Taking one of the samples in the data of gear-spalling conditions as an example, the effect of the ICEEMDAN decomposition signals is shown in Figure 7.

It can be seen from Figure 7 that PKO converged in generation 5 in the sample decomposition process, and the minimum envelope entropy was 8.2435. The sample signal was decomposed into 13 IMF components and 1 residual. The left-hand side of Figure 7b shows the time-domain waveform of the IMF component, and the right-hand side shows the spectral diagram of the corresponding IMF component. From the spectral diagram, it can be seen that, from IMF1 to IMF13, the components are arranged in sequence from high frequency to low frequency, and the components are concentrated near their respective central frequencies, effectively inhibiting the phenomenon of mode mixing. There are few irrelevant components, and the overall frequency separation effect of each component is good, while the decomposition efficiency is high, which is conducive to the post-processing of the bogie gearbox signals. Thus, the effectiveness and superiority of PKO-ICEEMDAN’s signal decomposition are illustrated.

### 3.4. Double Screening Criteria

Double screening criteria—correlation coefficient and variance contribution rate—were introduced to optimize the IMF components with rich fault information for signal reconstruction [32]. The correlation coefficient describes the degree of correlation between each IMF component and the original signals, while the variance contribution rate represents the ratio of IMF component variance to the original signals’ sequence variance. By setting the threshold, the useful IMF components are retained, and the redundant false IMF components are eliminated. The correlation coefficient *ρ*, variance contribution rate *λ,* and their unified threshold *TH* are calculated as follows:

(37) $$\rho =\frac{\sum_{i=1}^{n}\left[u_{i}-M(u)][v_{i}-M(v)\right]}{\sqrt{\sum_{i=1}^{n}{\left[u_{i}-M(u)\right]}^{2}{\left[v_{i}-M(v)\right]}^{2}}}$$

where *M* (*u*) and *M* (*v*) are the average of signals *u_i_* and *v_i_*, respectively, and *N* is the number of sampling points.

(38) $$\lambda_{k}=\frac{S_{k}^{2}}{S_{o}^{2}}$$

where *λ_k_* is the variance contribution rate of the *k*th-order IMF, $S_{k}^{2}$ is the variance of the *k*th-order IMF, and $S_{o}^{2}$ is the variance of the original signals

(39) $$TH=\frac{f_{\max}}{10\times {\left(f_{\max}\right)}^{-3}}$$

where *f*_max_ is the maximum value of the corresponding index.

**Figure 8:**
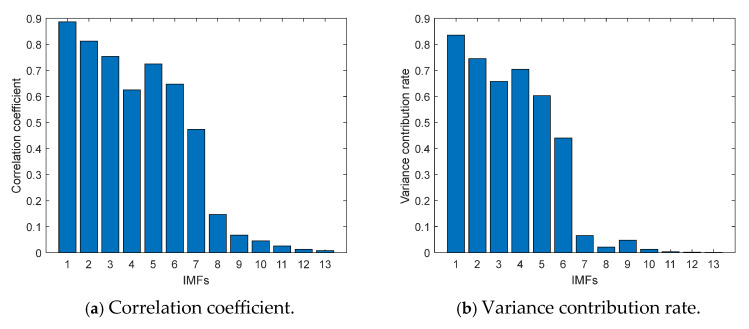
Correlation coefficient and variance contribution rate of signals.

Taking the above gear-spalling signals as an example, the correlation coefficient and variance contribution rate were calculated by using the above formulae, as shown in Figure 8.

**Figure 9:**
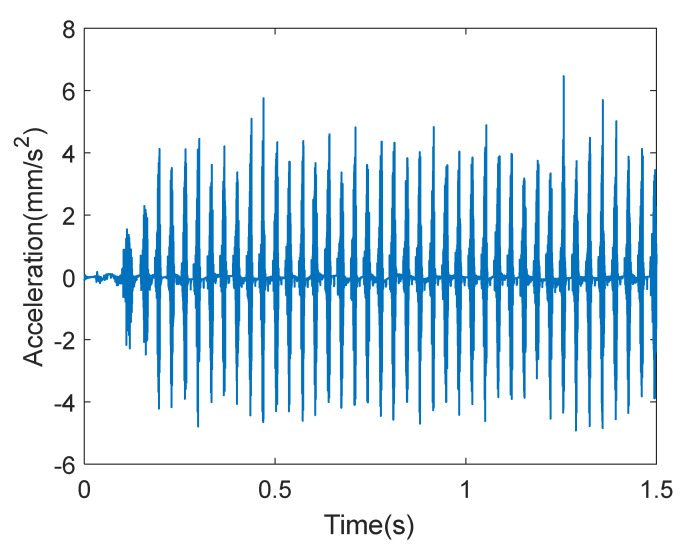
Time-domain waveform of reconstructed signals under spalling working condition.

The threshold value of the correlation coefficient was 0.1362, and the IMF components with correlation coefficients greater than 0.1362 were reserved. It can be seen from Figure 8a that the IMF1–IMF8 components were retained. The threshold value of the variance contribution rate was 0.0849, and the IMF components with a variance contribution rate greater than 0.0849 were reserved. It can be seen from Figure 8b that the IMF1–IMF6 components were retained. Considering them comprehensively, the IMF1–IMF8 components were retained for signal reconstruction in order to maximize the acquisition of fault information. The time-domain waveform of reconstructed signals of the sample described above is shown in Figure 9. Other sample data were processed in the same way and retained to the maximum-order IMF.

It can be seen from Figure 9 that the IMF components containing noise were removed from the reconstructed signals, and some impact components were more obvious. It follows that the reconstructed signals can highlight the core fault components more than the original signals.

### 3.5. Feature Extraction

**Figure 10:**
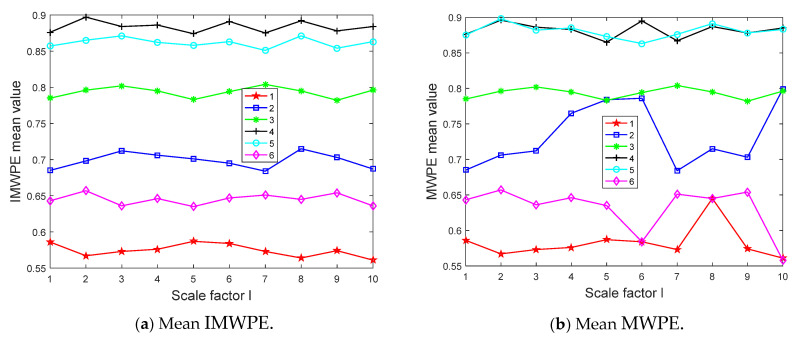
Mean IMWPE and MWPE of sample data under 6 working conditions.

IMWPE was extracted from the reconstructed signals, and MWPE was extracted at the same time for comparison. For the setting of relevant parameters, the time delay *τ* has a small impact on IMWPE, which is usually not studied in detail and generally takes a value of 1. For the embedding dimension *m*, if the value of *m* is too small, the reconstructed vector will become shorter. If the value of *m* is too large, it will not only increase the computational complexity but also fail to effectively reflect the subtle changes in the time series. Therefore, the range of the embedding dimension *m* is usually set to [3, 7]. There is no fixed standard for selecting the scale factor, and it is generally set to be greater than or equal to 10. Based on the theoretical research, we set *τ* = 1, *m* = 5, and *l* = 10 in this article. The mean IMWPE and MWPE of the sample data under 6 working conditions are shown in Figure 10 through calculation.

It can be seen from Figure 10 that the distribution of entropy extracted on different scale factors under different working conditions was relatively uniform. In contrast, IMWPE had a stable trend without aliasing, while MWPE had aliasing, which is not conducive to fault classification and identification in the later stage. It follows that IMWPE uses the improved multiple coarse-grained methods at the same scale factor as the feature vector of the classifier, which has more advantages than MWPE.

## 4. Fault Diagnosis and Comparative Experiments

### 4.1. Fault Diagnosis Based on SFOA-LSSVM

**Figure 11:**
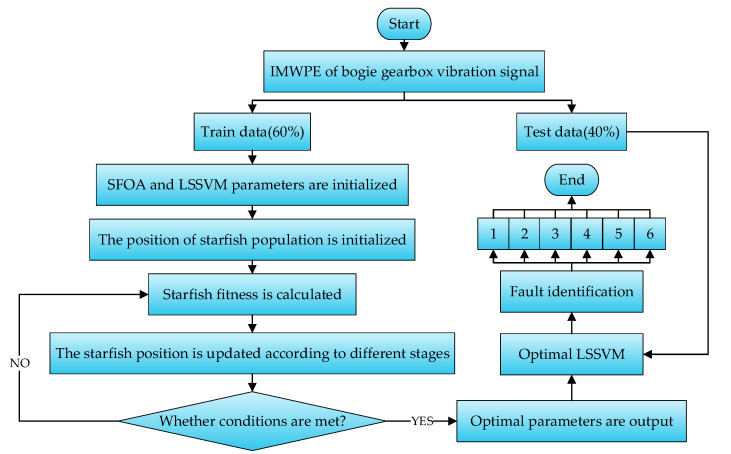
Fault diagnosis flowchart of SFOA-LSSVM.

LSSVM uses equality constraints instead of inequality constraints, making the calculation more efficient; however, the penalty factor *δ* and kernel function parameter *θ* need to be set manually. This model is prone to overfitting if the penalty factor is set too large; otherwise, it will lead to underfitting. The size of the kernel function parameter affects the size of the mapping space dimension, which is not conducive to fault classification. Therefore, the SFOA was used to optimize LSSVM by setting the fitness function. There are more advantages to this approach than to setting the parameters manually. The fault diagnosis flowchart of SFOA-LSSVM is shown in Figure 11.

It can be seen from Figure 11 that the process steps of SFOA-LSSVM fault diagnosis are as follows:

(1) The IMWPE of the vibration signals of the bogie gearbox is randomly divided into training data and testing data at a ratio of 6:4 as the input eigenvector of LSSVM.
(2) The population size of the SFOA is set to 30, and the maximum number of iterations is set to 100. The penalty factor *δ* and kernel function parameter *θ* in LSSVM are set to [0.01, 200].
(3) The position of the starfish population is initialized randomly.
(4) The mean square error of the LSSVM model corresponding to the individual position of each starfish is calculated as a fitness function.
(5) The position of the starfish population is updated according to the corresponding formula.
(6) Whether the iteration conditions are met is judged. The next iteration is performed if the conditions are not met. The optimization is stopped if the iteration conditions are met.
(7) The best parameter combination is output. The testing data are input into the LSSVM with the best combination of parameters for classification, and the fault diagnosis of the bogie gearbox is achieved.

**Figure 12:**
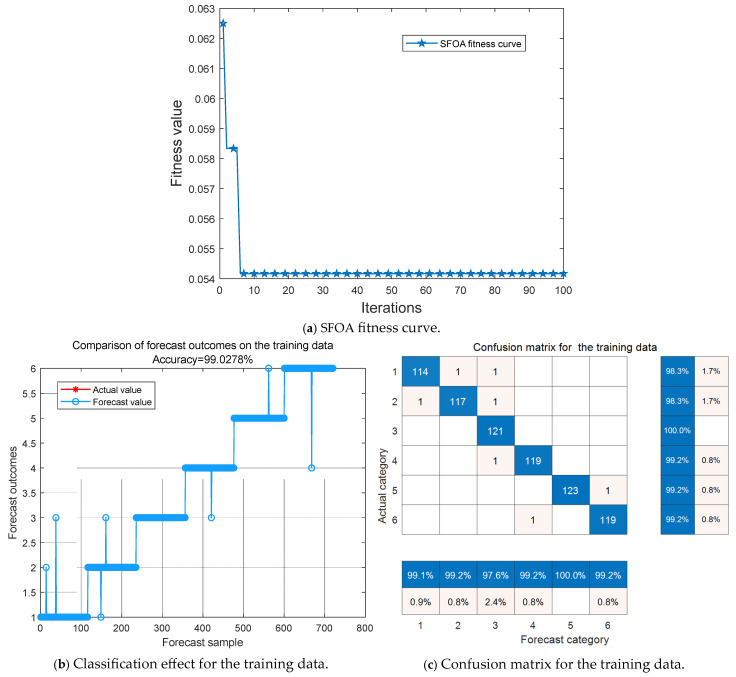

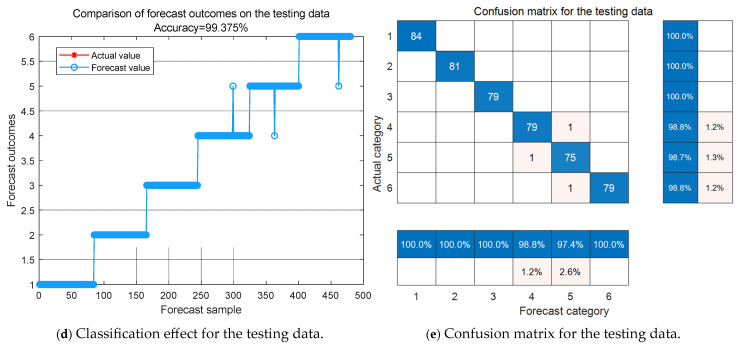
Simulation effect.

**Figure 13:**
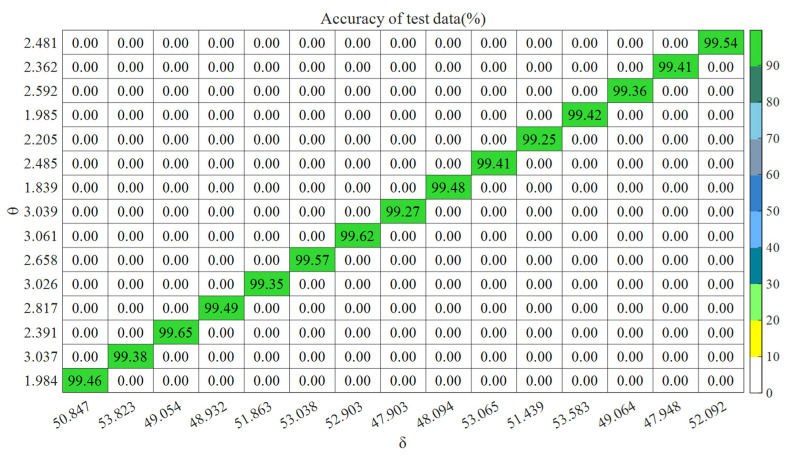
LSSVM parameter optimization heatmap (diagonal values are effective).

The model ran independently for 15 iterations, and the simulation effect of one iteration is shown in Figure 12. According to the accuracy of the testing data, the heatmap of the penalty factor and kernel function parameter of LSSVM is shown in Figure 13.

It can be seen from Figure 12 that the SFOA converged rapidly in the 6th generation and reached the minimum mean square error. The accuracy of these training data was 99.03%, and there were 7 prediction errors; the accuracy of the testing data was 99.38%, with only 3 prediction errors. It can be seen from Figure 13 that the model ran independently for 15 iterations, the optimal parameter region of *θ* was essentially concentrated in [1, 4], and the optimal parameter region of *δ* was essentially concentrated in [47, 54]. The accuracy of the testing data reached the highest value (99.65%) at the third iteration. At this time, the best parameter combination was [49.054, 2.391]. After sorting and calculation, the average accuracy of the training data was 99.13%, and the standard deviation was 0.09; the average accuracy of the testing data was 99.44%, and the standard deviation was 0.12. It follows that the model has a certain stability.

### 4.2. Comparative Experiments

In order to verify the superiority and effectiveness of the model proposed in this paper, the relevant variables were controlled, and comparative experiments were carried out using different signal decomposition methods, eigenvectors, classification algorithms, SFOA-LSSVM kernel functions, and various IMWPE parameters. Each model was run independently for 15 iterations. The indices of accuracy, macro-precision and macro-recall were introduced, and the average values of various indices were calculated to measure the advantages and disadvantages of these models.

#### 4.2.1. Comparative Experiment of Different Signal Decomposition Methods

The Fourier decomposition method (FDM), wavelet transform (WT), CEEMD, CEEMDAN, ICEEMDAN, and PKO-ICEEMDAN were used to decompose the vibration signals of the gearbox. The IMWPE of the reconstructed signals was obtained, and the SFOA-LSSVM model was used for fault diagnosis. The process of FDM noise reduction and reconstruction involves performing Fourier transform on the signals. The filter is used to retain useful components and eliminate noise components. Finally, the signals are reconstructed by inverse transformation. The process of WT noise reduction and reconstruction is as follows: (1) The vibration signals of the gearbox are decomposed into wavelet coefficients of different layers by the wavelet basis function. (2) The soft threshold function is adopted as the threshold function of wavelet threshold de-noising, and the fixed threshold criterion is selected to determine the wavelet threshold. (3) The processed wavelet coefficients are inversely transformed into the processed reconstructed signal. In addition to the above three performance indicators, time consumption indicators are also introduced. The statistics are based on the average values of various indicators in the testing data, as shown in Table 4.

It can be seen from Table 4 that the average values of the three indicators for fault diagnosis of the signals after PKO-ICEEMDAN decomposition were the highest, and the average time was the shortest, compared with other signal decomposition methods. This verifies the effectiveness of PKO-ICEEMDAN signal decomposition.

**Figure 14:**
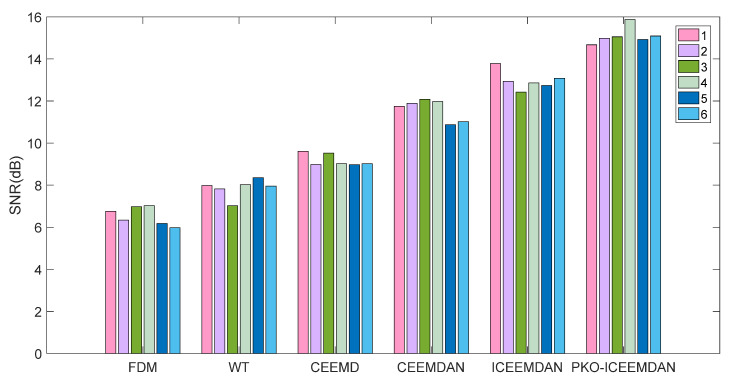
The average SNR with different signal decomposition methods.

In order to further verify the superiority of PKO-ICEEMDAN signal decomposition, the average signal-to-noise ratio (SNR) of the above decomposition method and reconstructed signals was calculated; the calculation results are shown in Figure 14.

It can be seen from Figure 14 that the average SNR of the signals processed by PKO-ICEEMDAN was the highest. Taking the normal gear working condition as an example, WT mode was 117% higher than FDM mode, CEEMD mode was 84% higher than WT mode, CEEMDAN mode was 53% higher than CEEMD mode, ICEEMDAN mode was 25% higher than CEEMDAN mode, and PKO-ICEEMDAN mode was 65% higher than ICEEMDAN mode. Therefore, it was verified that PKO-ICEEMDAN has more advantages in non-stationary signal decomposition through quantitative analysis.

#### 4.2.2. Comparative Experiment of Different Eigenvectors

The gearbox vibration signals were decomposed by PKO-ICEEMDAN. The PE, SE, FE, MPE, MSE, MFE, WPE, MWPE, and IMWPE of the reconstructed signals were extracted after the signals were reconstructed according to the double screening criteria, and the SFOA-LSSVM model was used for fault diagnosis. The statistics are based on the average values of various indicators in the testing data, as shown in Table 5.

It can be seen from Table 5 that the averages of the 3 indicators for fault diagnosis with IMWPE as the eigenvector were the highest compared with other eigenvectors; this verifies the effectiveness of IMWPE as a feature vector. Through further observation, it can be seen that the fault diagnosis index of multi-scale fault features was higher than that of single-scale features. Thus, the validity of multi-scale fault features was further verified.

#### 4.2.3. Comparative Experiment of Different Classification Algorithms

The gearbox vibration signals were decomposed by PKO-ICEEMDAN. The IMWPE of the reconstructed signals was extracted after the signals were reconstructed according to the double screening criteria. BP, SVM, LSTM, convolutional neural network (CNN), and LSSVM were used for fault diagnosis, and the SFOA was used to optimize them. The constructed BP neural network included an input layer, hidden layer, and output layer. The training process of SVM was essentially the same as that of LSSVM. For LSTM, the SFOA was used to optimize its learning rate, hidden layer nodes, and regularization coefficient. The forgetting gate outputs a value between 0 and 1 through the sigmoid function, indicating the forgetting degree of historical information. The input gate outputs a value between 0 and 1 through the sigmoid function, indicating the acceptance of new information. The output gate outputs a value between 0 and 1 through the sigmoid function, indicating the output degree of memory unit information. The fault diagnosis process of the CNN was as follows: (1) The model included one input layer, two convolution layers, two pooling layers, one fully connected layer, and one output layer. A shared convolution kernel and ReLU activation function were used. (2) The average pooling method was selected for the pooling layer. (3) The SFOA was adopted to optimize the learning rate of the CNN. Minimum envelope entropy was used as the fitness function. (4) The SFOA updated the position of starfish through continuous iteration. (5) If the termination conditions were met, the optimal parameters were output; otherwise, the next iteration was performed. (6) The feature data were flattened by the fully connected layer. (7) The output layer output the classification results through the softmax function. The statistics were based on the average values of various indicators in the testing data, as shown in Table 6.

It can be seen from Table 6 that the averages of the 3 indicators for fault diagnosis with SFOA-LSSVM as the classification algorithm were the highest compared with the other classification algorithms. This verifies the effectiveness of SFOA-LSSVM as a classification algorithm.

#### 4.2.4. Comparative Experiment of Different SFOA-LSSVM Kernel Functions

The gearbox vibration signals were decomposed by PKO-ICEEMDAN. The IMWPE of the reconstructed signals was extracted after the signals were reconstructed according to the double screening criteria. SFOA-LSSVM was used for fault diagnosis. Different kernel functions were set separately, including the linear kernel, polynomial kernel, tanh kernel, and RBF. At the same time, the comparative experiment of no kernel function was also carried out. The statistics were based on the average values of various indicators in the testing data, as shown in Table 7.

It can be seen from Table 7 that, compared with the other kernel functions, the average values of the 3 indicators of fault diagnosis with RBF as the kernel function were the highest. This verifies the effectiveness of RBF as the kernel function in this paper. The experiment without a kernel function shows that this classification is a nonlinear classification problem.

#### 4.2.5. Comparative Experiment of Different Values of IMWPE Parameters

**Figure 15:**
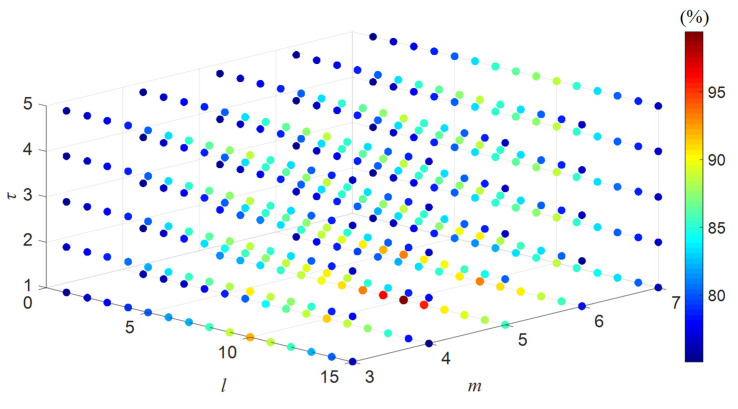
Three-dimensional schematic diagram of the average accuracy of testing data with different IMWPE parameters.

Parameter comparison experiments were carried out in order to verify the effectiveness of different values of IMWPE parameters. The value range of time delay *τ* in this paper is an integer between 1 and 5, the embedding dimension *m* is an integer between 3 and 7, and the scale factor *l* is an integer between 1 and 15. Different parameters of IMWPE were set, the fault diagnosis model proposed in this paper was run 15 times, and the average accuracy of the testing data was counted. The comparison results are shown in Figure 15.

It can be seen from Figure 15 that the average accuracy of the testing data reached the maximum when *l* = 10, *m* = 5, and *τ* = 1. The average accuracy decreased as the time delay increased. This is because much important frequency information will be lost and aliasing will occur if the time delay value is too large. The value of the embedding dimension cannot be too small or too large—the amount of important information in the reconstructed time series will be reduced if it is too small, while the calculation time will be too long to reveal the signal changes if it is too large. The accuracy will also be reduced if the scale factor is too large or too small. This is because it will lead to the problem of information redundancy if it is too large, while too small a value will result in incomplete fault information. Thus, the validity of the selected parameters of IMWPE in this paper was verified through comparative experiments.

## 5. Conclusions

ICEEMDAN was used to decompose the collected signals under six bogie gearbox working conditions in this study. PKO was used to optimize the white noise amplitude weight and noise addition times at the same time, and the optimal parameter combination was obtained. Thus, the problem of the unsatisfactory decomposition effect caused by manually determining parameters was avoided. The IMF components were optimized through the double screening criteria of correlation coefficient and variance contribution rate. The redundant IMF with a large amount of noise was eliminated for signal reconstruction; therefore, reconstructed signals with rich fault information were obtained. The IMWPE of the reconstructed signals was extracted as the feature vector of LSSVM, and the SFOA was used to optimize the parameters of LSSVM so as to achieve a more ideal diagnosis effect. The advantages of the proposed model in the field of fault diagnosis were verified compared with other diagnosis models. To better validate the practical applicability and generalization capability of the proposed method, the kernel function approach can be changed in practical applications, such as by combining multiple kernel functions or using adaptive kernel functions.

## Figures and Tables

**Table 1:** Six working conditions set in this paper.

Category Label	Working Condition of Large Helical Gear	Number of Samples
1	Normal	200
2	Crack	200
3	Spalling	200
4	Tooth-breaking	200
5	Scuffing	200
6	Plastic deformation	200
Total		1200

**Table 2:** Relevant gear parameters.

Parameters	Driving Small Helical Gear	Driven Large Helical Gear
Normal modulus (m)	0.005	0.005
Pressure angle (rad)	π/9	π/9
Addendum coefficient	1	1
Helix angle (rad)	π/15	π/15
Number of teeth	19	120
Tooth width (m)	0.075	0.07

**Table 3:** Optimal parameter combinations under various working conditions.

Category Label	*Nstd*	*NE*	Average Fitness
1	0.354	645	8.243
2	0.329	1025	8.312
3	0.297	947	8.349
4	0.416	562	8.436
5	0.348	687	8.819
6	0.402	742	8.235

**Table 4:** Averages of various indicators in testing data with different signal decomposition methods.

Decomposition Mode	Accuracy (%)	Macro-Precision (%)	Macro-Recall (%)	Time Consumption(s)
FDM	94.73	94.65	94.81	301.51
WT	95.51	95.62	95.46	298.34
CEEMD	95.78	95.39	95.16	297.36
CEEMDAN	97.45	97.72	97.48	291.81
ICEEMDAN	97.95	98.12	98.08	289.19
PKO-ICEEMDAN	99.44	99.46	99.52	275.94

**Table 5:** Averages of various indicators in testing data with different eigenvectors.

Different Eigenvectors	Accuracy (%)	Macro-Precision (%)	Macro-Recall (%)
PE	94.23	94.51	94.36
SE	94.56	94.31	94.72
FE	93.75	93.51	94.01
MPE	95.67	95.92	95.49
MSE	95.02	95.05	95.18
MFE	95.43	95.31	95.53
WPE	95.46	95.57	95.49
MWPE	96.92	97.03	97.06
IMWPE	99.44	99.46	99.52

**Table 6:** Averages of various indicators in testing data with different classification algorithms.

Classification Algorithms	Accuracy (%)	Macro-Precision (%)	Macro-Recall (%)
BP	95.21	95.36	95.28
SVM	95.89	95.91	95.76
LSTM	97.05	97.26	97.18
CNN	98.01	98.26	98.14
LSSVM	99.44	99.46	99.52

**Table 7:** Averages of various indicators in testing data with different SFOA-LSSVM kernel functions.

Different Kernel Functions	Accuracy (%)	Macro-Precision (%)	Macro-Recall (%)
No kernel function	60.65	59.95	60.34
Linear kernel	85.67	86.03	86.29
Polynomial kernel	90.37	90.02	90.46
Tanh kernel	93.56	93.41	93.19
RBF	99.44	99.46	99.52

## Data Availability

Data are contained within the article.

## Abbreviations

PKO	Pied kingfisher optimizer
IMF	Intrinsic mode function
EMD	Empirical mode decomposition
EEMD	Ensemble empirical mode decomposition
CEEMD	Complementary ensemble empirical mode decomposition
CEEMDAN	Complete ensemble empirical mode decomposition with adaptive noise
ICEEMDAN	Improved complete ensemble empirical mode decomposition with adaptive noise
SFOA	Starfish optimization algorithm
LSSVM	Least-squares support vector machine
FE	Fuzzy entropy
DE	Dispersion entropy
SE	Sample entropy
PSE	Power spectral entropy
PE	Permutation entropy
WPE	Weighted permutation entropy
MWPE	Multi-scale weighted permutation entropy
IMWPE	Improved multi-scale weighted permutation entropy
PNN	Probabilistic neural network
LSTM	Long short-term memory
RNN	Recurrent neural network
SVM	Support vector machine
MMI	Man–machine interface
HMI	Human–machine interface
PPM	Post-process module
VDS	Visual display system
*Nstd*	White noise amplitude weight
*NE*	Noise addition times
*δ*	Penalty factor
*θ*	Kernel function parameter
*m*	Embedding dimension
*τ*	Time delay
*l*	Scale factor
*S_l_*	The lower limit of the search space in PKO
*S_u_*	The upper limit of the search space in PKO
*N*	The population size
*M*	The problem dimension
*T_max_*	The maximum number of iterations
*ε*	Hunting ability
*η*	The control parameter
*q*	The flapping frequency of a pied kingfisher’s wings
*L_j_*	The lower limit of the *j*th dimensional design variable in SFOA
*U_j_*	The upper limit of the *j*th dimensional design variable in SFOA
*ξ*	The error amount
*ω*	Weight vector
*ρ*	Correlation coefficient
*λ*	Variance contribution rate
*TH*	Threshold

## References

[1] Xie J., Tang Y., Yang J., Wang T. (2023). Research on dynamic modeling and fault quantitative classification method of bogie gearbox. J. Cent. South Univ. (Sci. Technol.).

[2] Qin Y., Wang Y., Li Z.S., Wang B., Ding A., Wang C., Qin Y., Wang Y. (2025). An in-depth tutorial on BJTU-RAO bogie datasets for fault diagnosis. IEEE Access.

[3] Yang B., Wang T., Xie J., Yang J. (2023). Deep adversarial hybrid domain-adaptation network for varying working conditions fault diagnosis of high-speed train bogie. IEEE Trans. Instrum. Meas..

[4] Xie J., Cao S., Pan T., Wang T., Yang J., Chen J. (2025). A pruning-aware dynamic slimmable network using meta-gradients for high-speed train bogie bearing fault diagnosis. ISA Trans..

[5] Xue Y., Yang R., Chen X., Song B., Wang Z. (2025). Separable convolutional network-based fault diagnosis for high-speed train: A gossip strategy-based optimization approach. IEEE Trans. Ind. Inform..

[6] Zheng Z., Song D., Zhang W., Jia C. (2025). A fault diagnosis method for bogie axle box bearing based on sound-vibration multiple signal fusion. Appl. Acoust..

[7] Yuan B., Li Y., Chen S. (2025). Efficient gearbox fault diagnosis based on improved multi-scale CNN with lightweight convolutional attention. Sensors.

[8] Li K., Feng Z., Sun H., Shen K. (2021). Planetary gearbox fault diagnosis via extension EMD and GDE to identify instantaneous damping ratio. J. Vib. Shock.

[9] Xu Y., Wang H., Xu F., Bi S., Ye J. (2025). A sensor data-driven fault diagnosis method for automotive transmission gearboxes based on improved EEMD and CNN-BILSTM. Processes.

[10] Chen J., Zhou D., Lyu C., Lu C. (2017). An integrated method based on CEEMD-SampEn and the correlation analysis algorithm for the fault diagnosis of a gearbox under different working conditions. Mech. Syst. Signal Process..

[11] Zhu P., Liu Y., Liu Z., Chen J., Nie K. (2023). Fault diagnosis of synchronous generator rotating rectifier based on CEEMD and improved ELM. J. Beijing Univ. Aeronaut. Astronaut..

[12] Bouhalais M.L., Djebala A., Ouelaa N., Babouri M.K. (2018). CEEMDAN and OWMRA as a hybrid method for rolling bearing fault diagnosis under variable speed. Int. J. Adv. Manuf. Technol..

[13] Luan X., Tang J., Sha Y. (2024). Inter-shaft fault diagnosis method based on deep extreme learning machine optimized with dung beetle optimizer. J. Vib. Shock.

[14] Gao L., Gu Y., Chen C., Zhang P., Zhang Z. (2024). Wind turbine gearbox bearing fault diagnosis method based on ICEEMDAN and flexible wavelet threshold. J. Fail. Anal. Prev..

[15] Liu W., Wang Q., Xu F. (2024). Multi-sensor gearbox fault diagnosis using generalized minimum entropy deconvolution and main frequency center extraction. Meas. Sci. Technol..

[16] Hou S., Zheng J., Pan H., Feng K., Liu Q., Ni Q. (2024). Multivariate multi-scale cross-fuzzy entropy and SSA-SVM-based fault diagnosis method of gearbox. Meas. Sci. Technol..

[17] Wang X., Du Y., Ji X. (2024). Gearbox fault diagnosis based on adaptive variational mode decomposition–stationary wavelet transform and ensemble refined composite multiscale fluctuation dispersion entropy. Sensors.

[18] Li W., Wang F., Wang D. (2024). Fault diagnosis of planetary gearbox based on improved composite multi-scale sample entropy. J. Aerosp. Power.

[19] Yu X., Wang Y., Wang Y. (2025). Early fault diagnosis of gearbox teeth surface wear based on OSGMD-Hilbert envelope logarithmic analysis. J. Vib. Shock.

[20] Yin X., Mou Z., Wang Y. (2023). Fault diagnosis of wind turbine gearbox based on multiscale residual features and ECA-Stacked ResNet. IEEE Sens. J..

[21] Liang S., Ma J. (2021). Compound fault diagnosis of gearbox based on RLMD and SSA-PNN. Math. Probl. Eng..

[22] Wu P., Guo L., Duan Y., Zhou W., He G. (2019). Control loop performance monitoring based on weighted permutation entropy and control charts. Can. J. Chem. Eng..

[23] Jiang G., Xie P., Du S., Guo Y., He Q. (2017). A new fault diagnosis model for rotary machines based on MWPE and ELM. Insight Non-Destr. Test. Cond. Monit..

[24] Zhou C., Jia Y., Zhao S., Yang Q., Liu Y., Zhang Z., Wang T. (2023). A mechanical part fault diagnosis method based on improved multiscale weighted permutation entropy and multiclass LSTSVM. Meas. J. Int. Meas. Confed..

[25] Liu B., Cai J. (2025). A new fault diagnosis of rolling bearing using RCMPNDE and SSA-PNN. Ind. Lubr. Tribol..

[26] Chen C., Ren X., Cheng G. (2024). Research on distributed fault diagnosis model of elevator based on PCA-LSTM. Algorithms.

[27] Balamurugan A., Shunmugakani S., Ramya R., Saravanan S. (2024). Fault diagnosis of three-phase induction motor (IM) using a hybrid ELSE-RNN technique. IETE J. Res..

[28] Wang B., Qiu W., Hu X., Wang W. (2024). A rolling bearing fault diagnosis technique based on recurrence quantification analysis and Bayesian optimization SVM. Appl. Soft Comput..

[29] Yan X., Hua X., Jiang D., Xiang L. (2024). A novel robust intelligent fault diagnosis method for rolling bearings based on SPAVMD and WOA-LSSVM under noisy conditions. Meas. Sci. Technol..

[30] Bouaouda A., Hashim F.A., Sayouti Y., Hussien A.G. (2024). Pied kingfisher optimizer: A new bio-inspired algorithm for solving numerical optimization and industrial engineering problems. Neural Comput. Appl..

[31] Zhong C., Li G., Meng Z., Li H., Yildiz A.R., Mirjalili S. (2025). Starfish optimization algorithm (SFOA): A bio-inspired metaheuristic algorithm for global optimization compared with 100 optimizers. Neural Comput. Appl..

[32] Li M., He Y., Ma D., Tang Q., Hu M. (2022). Rolling bearing fault diagnosis based on ICEEMDAN-MPE and AO-LSSVM. Electron. Meas. Technol..

